# Corrosion Resistance of Shape Recoverable Fe-17Mn-5Si-5Cr Alloy in Concrete Structures

**DOI:** 10.3390/ma13235531

**Published:** 2020-12-04

**Authors:** Jaehoon Joo, Minjoo Kang, Dongmin Shin, Eunhye Seo, Dohyung Kim, Yeongmo Yeon, Kinam Hong, Wookjin Lee, Junghoon Lee

**Affiliations:** 1 Department of Metallurgical Engineering, Pukyong National University, 45, Yongso-ro, Nam-Gu, Busan 48513, Korea; jjh04@pukyong.ac.kr (J.J.); minju2930@pukyong.ac.kr (M.K.); 201511620@pukyong.ac.kr (D.S.); seh9067@pukyong.ac.kr (E.S.); 2 Dongnam Regional Division, Korea Institute of Industrial Technology (KITECH), Yangsan 50623, Korea; dhyungkim@kitech.re.kr (D.K.); wkjinlee@kitech.re.kr (W.L.); 3 Department of Civil Engineering, Chungbuk National University, 1 Chungdae-ro, Seowon-Gu, Cheongju, Chungbuk 28644, Korea; yym235@chungbuk.ac.kr (Y.Y.); hong@chungbuk.ac.kr (K.H.)

**Keywords:** shape memory alloy, ferrous alloy, corrosion, concrete

## Abstract

The shape memory effect of steel (i.e., Fe-Mn-Si alloys) enables the tensile strengthening of concrete against tensile stress and unexpected structural vibrations. For practical application, the corrosion resistance of shape-memorable Fe-based steel should be verified. In this study, the corrosion resistance of an Fe-based (Fe-16Mn-5Si-4Ni-5Cr-0.3C-1Ti) shape memory alloy (FSMA), a promising candidate for concrete reinforcement, was investigated by comparing it with general carbon steel (S400). The corrosion resistance of FSMA and S400 inserted in a cement mortar was evaluated using electrochemical methods. FSMA has a more stable passive oxide layer in aqueous solutions with various pH values. Thus, the corrosion resistance of the FSMA sample was much higher than that of the S400 carbon steel, which has a passivation layer in strongly alkaline solution. This stable oxide layer reduced the sensitivity of the corrosion resistance of FSMA to changes in the pH, compared to S400. Furthermore, owing to the stable passive oxide layer, FSMA exhibited a higher corrosion resistance in concrete and a lower decrease in corrosion resistance because of the neutralization of concrete. Therefore, FSMA is a promising candidate for providing reinforcement and reparability, resulting in stable and durable concrete.

## 1. Introduction

Considering their functionalities, shape memory alloys exhibiting superelasticity and shape recovery effects have been widely investigated for applications. It is well known that recovery of the original shape is caused by a phase transformation from martensite, formed by a stress-induced transformation, to austenite under heating [1,2,3]. Ti-Ni-based alloys have high recoverability of their initial shape at relatively low temperatures [4,5,6,7,8]. However, the properties of Ti-Ni-based alloys are very sensitive to the chemical composition of the alloying elements. In addition, owing to cost issues, Ti-Ni-based alloys are rarely considered as structural materials. Therefore, the shape recovery effects of various alloys have been investigated to extend the application fields. Since a single crystal of Fe-30Mn-Si alloy has been reported to have ~9% shape recovery, Fe-based alloys have attracted various research fields owing to their relatively low cost. In particular, Fe-based alloys showing the shape memory effect can be employed in structural construction and reinforcement of damaged structures [9,10].

Generally, concrete structures composed of cement-based composites and rebars have been used as a core material for construction. Cement-based composite with sand and gravel has excellent stability under compressive stress, whereas it is relatively weak under tensile conditions. Therefore, a rebar with a relatively high tensile strength is used as a reinforcement for cement-based composites. Prestressed concrete, which has a compressive stress, is employed to reduce the damage to concrete structures under tensile stress. The compressive stress in concrete can be induced by the tendon elongated in the elastic region. Therefore, with respect to the size of the concrete structure, various equipment and spaces for prestressing are required [11,12,13,14,15,16]. However, the use of such equipment has disadvantages, in that the construction process is complicated and the construction period is increased.

Since Fe-based shape memory alloys can cause a compressive recovery stress of 200–500 MPa, they can be applied to prestressed concrete. Recently, Fe-Mn-Si-based alloys have been studied for potential applications in prestressed concrete. For instance, Dong et al. [17] demonstrated that the Fe-17Mn-5Si-10Cr-4Ni-1(V,C) (wt.%) alloy can generate a compressive stress of up to 400 MPa via shape recovery in the restrained condition while heating the alloy to 220 °C. Later, Lee et al. [14] demonstrated that the same alloy can induce a compressive stress of approximately 400 MPa by heating it to 160 °C (i.e., the maximum allowable temperature of concretes). The prestressing of concrete beams by the alloy has been demonstrated by Shahverdi et al. [18]. In this regard, Fe-17Mn-5Si-5Cr containing 1 Ti and 0.3 C (wt.%) exhibited a high recovery stress, close to 450 MPa [19].

Even though the recoverable mechanical properties of Fe-Mn-Si-based shape memory alloys were investigated, the stability of this alloy should be explored for actual application as a concrete reinforcement. To confirm the stability of the Fe-17Mn-5Si-5Cr alloy in concrete environments, we evaluated the corrosion properties by comparing them with general carbon steel (S400), which is typically used for reinforcing concrete. The corrosion properties were evaluated in NaCl solutions with various pH values while reinforcement metals were mounted in a mortar, considering the application conditions.

## 2. Materials and Methods

An Fe-17Mn-5Si-5Cr shape memory alloy (FSMA) containing 1 Ti and 0.3 C [19] was prepared via vacuum induction melting, followed by hot rolling. First, a 50 kg ingot of the alloy was cast, and a homogenization heat treatment was given for 6 h at 1250 °C. Afterward, the ingot was forged and hot-rolled at 1000 °C to 5 mm.

**Figure 1:**
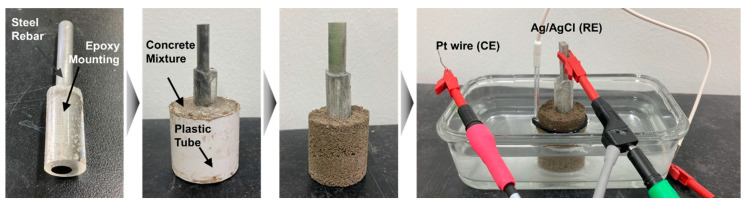
The preparation procedure of the sample and the electrochemical cell (i.e., reference electrode (RE) and counter electrode (CE)) for the corrosion test.

The chemical compositions of FSMA and S400 are summarized in Table 1. The FSMA andS400 samples were mounted in epoxy resin to expose a specific area to a corrosive environment. The exposed surfaces of the FSMA and the S400 were ground with sandpaper (grit #400). Then, the mounted samples were placed in a mixed mortar paste. The mortar was solidified at room temperature for 24 h and cured for 30 days. Figure 1 shows the sample setup with the epoxy resin mounting and the mortar.

An aqueous solution of 3.5 wt.% NaCl was used for the electrochemical corrosion test. Considering the concrete environment, the pH of the salt solution was adjusted with CaO. The corrosion resistance of the FSMA and the S400 in the concrete structure were evaluated with a potentiodynamic polarization test using a potentiostat at room temperature (VersaSTAT 4, Princeton Applied Research, Oak Ridge, TN, USA). Ag/AgCl and Pt wires were used as the reference and counter electrodes, respectively. Figure 1 shows the test setup of the cell arrangement. The potential was scanned from −400 to 800 mV vs. open circuit potential with a rate of 2 mV/s. The test conditions are summarized in Table 2. To minimize experimental errors in electrochemical corrosion characterizations, seven samples were tested in each condition. Next, the free corrosion potential and free corrosion current density were estimated by Tafel fitting of potentiodynamic polarization curves, which are the results of the electrochemical corrosion test [20,21,22]. Excluding the maximum and minimum free corrosion current densities, five values were averaged. The potentiodynamic polarization curve, which has the most similar value to the average, was selected and is shown here as a representative result.

The surface chemical composition and oxide state were analyzed using X-ray photoelectron spectroscopy (XPS, Thermo Fisher Scientific, Greenwich, UK). In addition, the microstructures of the FSMA and S400 samples were observed using a field-emission scanning electron microscope (FE-SEM, Jeol, Tokyo, Japan).

## 3. Results and Discussion

### 3.1. Microstructure of Test Sample

**Figure 2:**
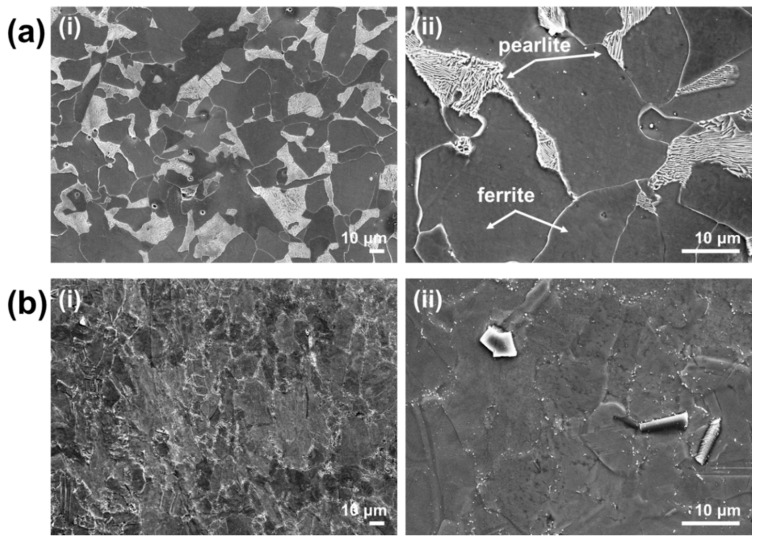
SEM images of the microstructures of (**a**) S400 and (**b**) Fe-based shape memory alloy (FSMA). (**i**) and (**ii**) are the images at low (×500) and high (×2000) magnifications, respectively.

Figure 2 shows the microstructure of the S400 and FSMA samples observed using FE-SEM. Figure 2a shows the microstructure of the S400, depicting a typical mixed microstructure of ferrite and pearlite. The microstructure of the FSMA shows equiaxed grain structures containing precipitates, as shown in Figure 2b. The microstructure of this alloy consists mainly of austenite, and the precipitates are TiC phases. It was reported that FSMA exhibits a yield strength as high as 400 MPa in the heat-treated solution and a high mechanical strength while the shape is recovered; so, application to functional components in prestressed civil engineering structures is possible [23].

### 3.2. Corrosion Test without Concrete Preparation

**Figure 3:**
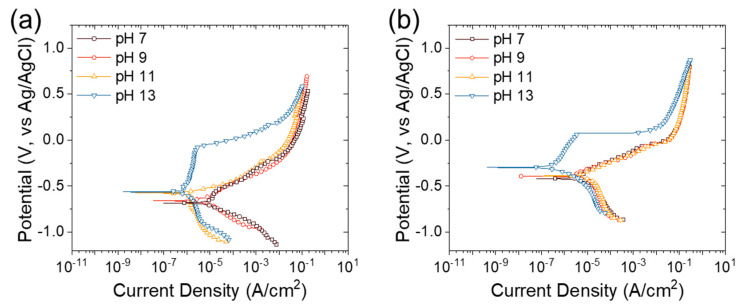
Potentiodynamic polarization curves of (**a**) S400 and (**b**) Fe-based shape memory alloy (FSMA) in 3.5 wt.% NaCl solution with various pH values, adjusted by CaO.

To compare the corrosion resistance of S400 and FSMA, the samples with epoxy mounting were tested by potentiodynamic polarization in a 3.5 wt.% NaCl solution with pH values varying from 7 to 13, adjusted by adding CaO, considering the concrete condition. Figure 3 shows the potentiodynamic polarization curves. Table 3 summarizes the free corrosion potential, free corrosion current density, and polarization resistance. The polarization resistance is directly related to the corrosion resistance of metals. The free corrosion current density of the S400 decreased with the increase in the pH because of the stabilized surface oxide layer of iron in the alkaline environment. Thus, the polarization resistance also increased with the pH of the NaCl solution. The free corrosion current density of the FSMA also decreased with the pH, such that the maximum polarization resistance of the FSMA was shown in the pH 13 solution. These are the general corrosion behaviors of iron-based alloys, which have a passive oxide layer in an alkaline solution [24,25,26,27]. Nevertheless, it should be noted that the polarization resistance of the FSMA was greater than that of the S400 by 4.7-fold at a pH of 7, 1.5-fold at a pH of 9, 2.1-fold at a pH of 11, and 1.5-fold at a pH of 13. These results indicate that the FSMA has higher corrosion resistance in corrosive solutions with a wider pH range than S400 carbon steel. Moreover, the differences in the free corrosion current density and polarization resistance of the S400 and the FSMA were more significant in lower pH solutions. Therefore, the corrosion resistance of S400 is more sensitive to a decrease in pH than the corrosion resistance of FSMA.

### 3.3. Passive Oxide Layer

**Figure 4:**
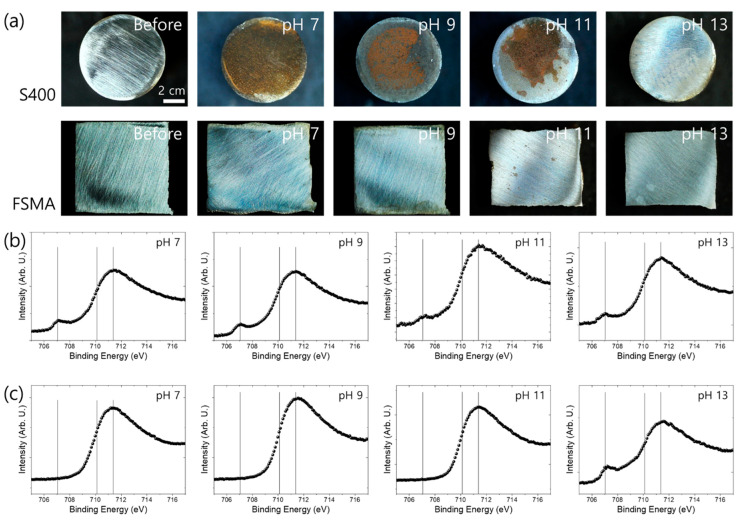
(**a**) Optical images of S400 and Fe-based shape memory alloy immersed in deionized water for a day with various pH values, adjusted by CaO. XPS core-level spectra of Fe 2p3/2 of (**b**) FSMA and (**c**) S400 immersed in deionized water for a day with various pH values, adjusted by CaO.

The surface oxide layers of the S400 and the FSMA were expected to affect the corrosion resistance. Thus, XPS analysis was conducted after immersion of the sample for a day in deionized water with various pH values adjusted by CaO. Figure 4a shows the surface images of the sample before and after immersion in water for a day at various pH values. Significant iron rust was found on the surface of the S400 immersed in water with pH values of 7, 9, and 11, indicating that the corrosion resistance of S400 is not good for a pH of 7–11. However, the sample immersed in the pH 13 solution showed no rust on the surface. This result is in strong agreement with the finding that the polarization resistance of the S400 was significantly enhanced in the pH 13 solution. For the FSMA, rust, a product of iron corrosion, was not found on the surface, indicating that FSMA has a more stable passive oxide layer, enhancing the corrosion resistance compared to S400 carbon steel. Figure 4b,c shows the XPS core-level spectra of the Fe 2p3/2 of the S400 and the FSMA shown in Figure 4a. The peak at ~707.0 eV corresponds to the Fe^0^, indicating metal bonding [28,29]. The peaks at ~710.1 eV and ~711.4 eV correspond to iron oxide and iron hydroxide, respectively [28,29]. Therefore, because the rust was also composed of oxide and hydroxide, the oxide forming a passive layer cannot be distinguished in the Fe 2p spectra. However, because the stable passive oxide layer was thin enough to reflect the signal of the base metal, the Fe^0^ metal peak can be found for the surfaces without rust.

**Figure 5:**
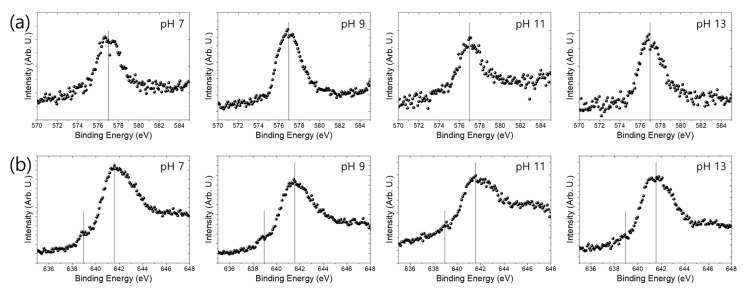
XPS core-level spectra of (**a**) Cr 2p3/2 and (**b**) Mn 2p3/2 of Fe-based shape memory alloy immersed in deionized water for a day with various pH values, adjusted by CaO.

**Figure 6:**
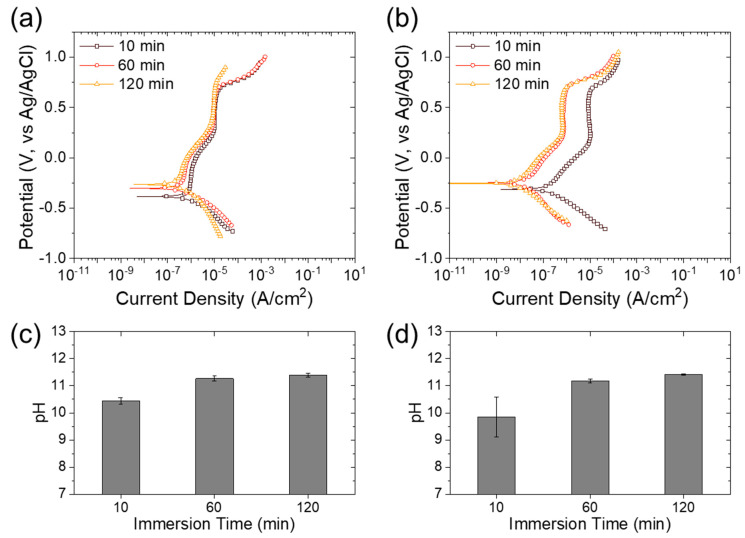
Potentiodynamic polarization curves of (**a**) S400 and (**b**) Fe-based shape memory alloy inserted in concrete in a 3.5 wt.% NaCl solution with various short-term immersions. pH of a 3.5 wt.% NaCl solution immersed for 120 min with (**c**) S400- and (**d**) FSMA-inserted concrete.

Even though the S400 contains a small amount of Mn as an alloying element, no signal for Mn was found in the XPS spectra. In addition, Cr was not included in the S400. Therefore, further analysis of the XPS core-level spectra of Cr 2p and Mn 2p was conducted for the FSMA (Figure 5). The peak at ~577 eV in the XPS core-level spectra of Cr 2p3/2 corresponds to Cr^3+^, indicating Cr_2_O_3_, Cr(OH)_3_, and CrOOH [30]. This peak can be found in the Cr 2p3/2 spectra (Figure 6a) of the FSMA immersed in the pH range of 7–13. These oxides and hydroxides are well known to improve the passivity of iron-based alloys. In addition, a peak corresponding to manganese metal (Mn^0^) can be found at ~639 eV in the Mn 2p3/2 spectra (Figure 6b) of the FSMA immersed in the pH range of 7–13 [30]. However, even though the intensity of the Mn^0^ peak is not significant, the presence of the Mn^0^ peak indicates that the thickness of the oxide layer was thin enough to detect the base metal in the XPS analysis. In addition, a peak corresponding to manganese oxides and hydroxide was detected at ~641.6 eV [30]. These results indicate that the hydroxides and oxides of chromium and manganese were incorporated with iron oxide and hydroxide on the FSMA in aqueous solution. Such oxide and hydroxide structures provide more stability in aqueous solutions [31,32,33,34]. Thus, FSMA exhibited better corrosion resistance than S400 carbon steel.

### 3.4. Short-Term Corrosion Test with Concrete Preparation

Figure 6a,b shows the potentiodynamic polarization curves of the rebar materials (S400 and FSMA) in mortar. Table 4 summarizes the free corrosion potential, the free corrosion current density, and the polarization resistance estimated from the potentiodynamic polarization curves in Figure 6. The cement is composed of CaO and, hence, the mortar pH increased when it was wetted by water. After dipping the mortar sample (cement and epoxy-mounted metals (S400 and FSMA)) in the NaCl solution, the pH of the solution was measured (Figure 6c,d). The increase in the dipping time of the mortar sample decreased the free corrosion current density and increased the polarization resistance. The polarization resistance of the S400 was increased by 2.1- and 1.7-fold for the increase in dipping time from 10 to 60 min and from 60 to 120 min, respectively. The FSMA also showed a significant increase in polarization resistance with an immersion time in NaCl solution, such as 8.4- and 1.2-fold for the increase in dipping time from 10 to 60 min and from 60 to 120 min, respectively. Such an increase in the polarization resistance (i.e., corrosion resistance) of rebar metals is attributed to the dissolution of CaO, which increased the pH by creating more OH^-^ ions (i.e., CaO + H_2_O → Ca^2+^ + 2OH^-^). The dissolved CaO increased the pH of the concrete and the concrete with a high pH also increased the pH of the corrosive solution (Figure 6c,d). The increase in the pH of the corrosive solution for 120 min indicates an increase in the pH of the mortar. Therefore, the iron-based alloys passivated in alkaline environments are more stable with an increased dipping time in aqueous solutions, and thus have a higher corrosion resistance [24,26,27]. The differences in the free corrosion current density and the polarization resistance of the S400 and the FSMA were more significant with increased immersion time in the NaCl solution, which was also observed for the test without mortar in various pH solutions (Figure 3). These results indicate that FSMA has a more stable surface passivation layer, which enhances its corrosion resistance.

### 3.5. Long-Term Corrosion Test with Mortar Preparation 

**Figure 7:**
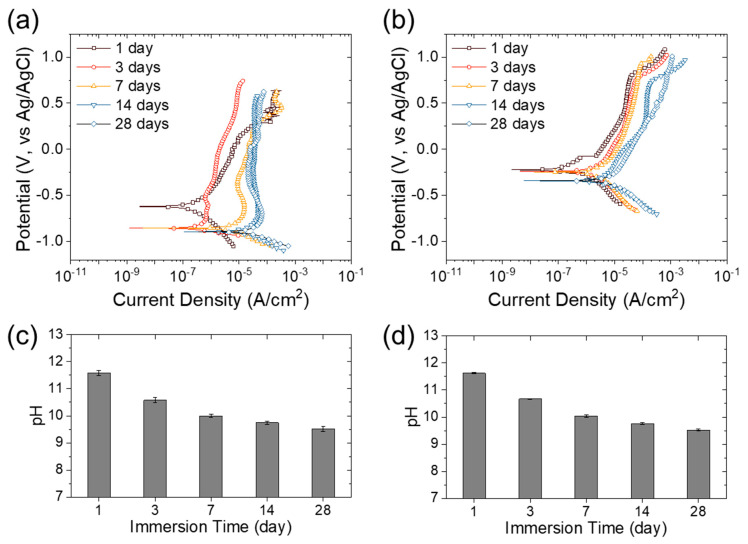
Potentiodynamic polarization curves of (**a**) S400 and (**b**) Fe-based shape memory alloy (FSMA) inserted in mortar in a 3.5 wt.% NaCl solution with various long-term immersion durations. pH of 3.5 wt.% NaCl solutions in which (**c**) S400- and (**d**) FSMA-inserted mortar was immersed for 28 days.

Figure 7a,b shows the potentiodynamic polarization curves of the rebar material (S400 and FSMA) in the mortar immersed in a 3.5 wt.% NaCl solution for up to 28 days. Table 5 summarizes the free corrosion potential, free corrosion current density, and polarization resistance estimated from the potentiodynamic polarization curves in Figure 7. In addition, the pH change during the immersion of the mortar in the NaCl solution is shown in Figure 7c,d. In contrast to the short-term immersion (shown in Figure 6, exhibiting an increase in pH of up to ~11.5), the pH of the NaCl solution gradually decreased from a pH of 11.5 to 9.5 with the increase in immersion time after one day. There was no difference in the pH change between the S400 and the FSMA. This decrease in pH was due to the dissolution of CO_2_ gas neutralizing the alkaline NaCl solution. However, the CaO in the mortar was continuously dissolved by the absorbed water in the concrete, and the OH^-^ ions maintained diffusion out of the corrosive solution. Therefore, the decrease in the pH of the NaCl solution indicates a decrease in the pH of the mortar. Such a change in the pH of the concrete affects the corrosion resistance of rebar metals (S400 and FSMA). A higher pH is effective for enhancing the corrosion resistance of iron-based alloys by forming a passivation layer. However, since the pH inside the concrete block was lower than the pH of the leachate, the pH of the mortar decreased with immersion time for more than a day. Because of this decrease in pH inside the concrete block, the free corrosion current density increased, and the polarization resistance significantly decreased. Owing to the immersion of mortar in the aqueous solution for 28 days, the polarization resistance of the S400 decreased to 1.0 Ω∙cm^2^, which is only ~0.4% of the polarization resistance for immersion for a day. The FSMA also showed a significant decrease in polarization resistance after immersion for 28 days—9.6 Ω∙cm^2^, which is ~2.8% of the polarization resistance for immersion for a day. Nevertheless, the FSMA showed a lower rate of corrosion resistance and a higher corrosion resistance than the S400. These results indicate that the corrosion resistance of FSMA is less sensitive to the change in pH compared to S400 carbon steel. In addition, the polarization resistance of the FSMA in the concrete was greater than that of the S400 by 1.5-, 1.7-, 5.8-, 13.4-, and 9.6-fold for the immersion in salt water for 1, 3, 7, 14, and 28 days, respectively. These results suggest that FSMA has higher corrosion resistance in a concrete environment than S400 carbon steel.

## 4. Conclusions

The corrosion resistance of the Fe-17Mn-5Si-5Cr alloy, which has a shape memory property enabling application to the reinforcement of prestressed concrete, was investigated by comparing it to general S400 carbon steel. Based on the experimental results, the following conclusions were reached:

1. Owing to the Mn and Cr, the shape-memorable Fe-17Mn-5Si-5Cr alloy forms a stable passivation oxide layer in alkaline environments, which has a similar pH to concrete.
2. This passivation oxide layer enabled the Fe-17Mn-5Si-5Cr alloy to obtain a higher corrosion resistance than S400 carbon steel in salt water (more than 140% in the pH range of 7–13).
3. Because of the dipping in salt water for 28 days, the corrosion resistance of the Fe-17Mn-5Si-5Cr alloy decreased by 97.2%, whereas that of the S400 decreased by 99.6%, indicating a lower corrosion sensitivity of the Fe-17Mn-5Si-5Cr alloy to the change in mortar pH.
4. The Fe-17Mn-5Si-5Cr alloy showed a higher corrosion resistance than the S400 (more than 150% in the mortar), indicating better chemical stability in the concrete structure.
5. These results indicate that the Fe-17Mn-5Si-5Cr shape memory alloy exhibits a higher corrosion resistance than S400 carbon steel (commonly used for reinforcing concrete) and that it is a potential candidate for fabricating structures based on prestressed concrete and reinforced concrete.

## Figures and Tables

**Table 1:** Chemical composition (in wt.%) of S400 and FSMA ^1^.

Name	Fe	Mn	Si	Cr	Ni	Ti	C
S400	Bal.	<1.0	-	-	-	-	0.2
FSMA	Bal.	17	5	5	4	1	0.3

^1^ Fe-based shape memory alloy.

**Table 2:** Test conditions for corrosion resistance of FSMA ^1^ and S400 in concrete.

Reinforcement Material	Mortar	pH Adjustment with CaO	Short-Term Immersion Time	Long-Term Immersion Time
S400	Ⅹ	○	30 min	-
	○	Ⅹ	10, 60, 120 min	1, 3, 7, 14, 28 days
FSMA	Ⅹ	○	30 min	-
	○	Ⅹ	10, 60, 120 min	1, 3, 7, 14, 28 days

Note: ^1^ Fe-based shape memory alloy; Ⅹ: not applicable; ○: applicable

**Table 3:** Estimated free corrosion potential (E_corr_), free corrosion current density (i_corr_), and polarization resistance (R_p_) of S400 and Fe-based shape memory alloy (FSMA) from the potentiodynamic polarization curves in Figure 3.

Sample	pH	E_corr_ (V vs. Ag/AgCl)	i_corr_ (A/cm^2^)	R_p_ (kΩ∙cm^2^)
S400	7	−0.685	1.7 × 10^−5^	1.8
	9	−0.659	4.3 × 10^−6^	6.8
	11	−0.575	2.2 × 10^−6^	10.9
	13	−0.558	7.7 × 10^−7^	76.4
FSMA	7	−0.412	4.7 × 10^−6^	8.7
	9	−0.392	3.2 × 10^−6^	11.8
	11	−0.387	1.6 × 10^−6^	23.2
	13	−0.288	3.8 × 10^−7^	111.2

**Table 4:** Estimated free corrosion potential (E_corr_), free corrosion current density (i_corr_), and polarization resistance (R_p_) from the potentiodynamic polarization curves in Figure 6.

Sample	Immersion Time	E_corr_ (V vs. Ag/AgCl)	i_corr_ (A/cm^2^)	R_p_ (kΩ∙cm^2^)
S400	10 min	−0.379	6.5 × 10^−7^	72.6
	60 min	−0.305	2.3 × 10^−7^	154.7
	120 min	−0.255	2.2 × 10^−7^	267.9
FSMA	10 min	−0.312	1.9 × 10^−7^	261.1
	60 min	−0.246	3.0 × 10^−8^	2107.1
	120 min	−0.253	2.9 × 10^−8^	2118.2

**Table 5:** Estimated free corrosion potential (E_corr_), free corrosion current density (i_corr_), and polarization resistance (R_p_) of S400 and Fe-based shape memory alloy from the potentiodynamic polarization curves in Figure 7.

Sample	Immersion Time	E_corr_ (V vs. Ag/AgCl)	i_corr_ (A/cm^2^)	R_p_ (kΩ∙cm^2^)
S400	1 day	−0.621	3.3 × 10^−7^	227.4
	3 day	−0.856	5.2 × 10^−7^	47.4
	7 day	−0.858	9.2 × 10^−6^	6.4
	14 day	−0.895	1.3 × 10^−5^	4.1
	28 day	−0.895	3.7 × 10^−5^	1.0
FSMA	1 day	−0.231	2.2 × 10^−7^	350.1
	3 day	−0.241	1.3 × 10^−6^	81.9
	7 day	−0.248	3.5 × 10^−6^	37.1
	14 day	−0.342	5.0 × 10^−6^	22.0
	28 day	−0.341	6.9 × 10^−6^	9.6

## References

[1] Dong Z., Kajiwara S., Kikuchi T., Sawaguchi T. (2005). Effect of pre-deformation at room temperature on shape memory properties of stainless type Fe–15Mn–5Si–9Cr–5Ni–(0.5–1.5) NbC alloys. Acta Mater..

[2] Otsuka H., Yamada H., Tanahashi H., Maruyama T. (1990). Shape memory effect in Fe-Mn-Si-Cr-Ni polycrystalline alloys. Mater. Sci. Forum.

[3] Sato A., Yamaji Y., Mori T. (1986). Physical properties controlling shape memory effect in Fe-Mn-Si alloys. Acta Metall..

[4] Kim J.I., Hwang K.S. (2019). Effect of Annealing Temperature on the Shape Memory Properties of Ti-44.5 Ni-5Cu and Ti-45.2 Ni-5Cu (at%) Alloys. Korean J. Met. Mater..

[5] Liu Y. (2015). The superelastic anisotropy in a NiTi shape memory alloy thin sheet. Acta Mater..

[6] Miyazaki S., Otsuka K., Suzuki Y. (1981). Transformation pseudoelasticity and deformation behavior in a Ti-50.6 at% Ni alloy. Scr. Metall..

[7] Narayana P., Kim S.-W., Hong J.-K., Reddy N., Yeom J.-T. (2018). Estimation of transformation temperatures in Ti–Ni–Pd shape memory alloys. Met. Mater. Int..

[8] Nishida M., Wayman C.M., Honma T. (1986). Precipitation processes in near-equiatomic TiNi shape memory alloys. Metall. Trans. A.

[9] Hong K., Lee S., Han S., Yeon Y. (2018). Evaluation of Fe-based shape memory alloy (Fe-SMA) as strengthening material for reinforced concrete structures. Appl. Sci..

[10] Lagoudas D.C. (2008). Shape Memory Alloys: Modeling and Engineering Applications.

[11] Arabi-Hashemi A., Lee W., Leinenbach C. (2018). Recovery stress formation in FeMnSi based shape memory alloys: Impact of precipitates, texture and grain size. Mater. Des..

[12] Choi E., Chung Y.-S., Choi J.-H., Kim H.-T., Lee H. (2010). The confining effectiveness of NiTiNb and NiTi SMA wire jackets for concrete. Smart Mater. Struct..

[13] Czaderski C., Hahnebach B., Motavalli M. (2006). RC beam with variable stiffness and strength. Constr. Build. Mater..

[14] Lee W., Weber B., Feltrin G., Czaderski C., Motavalli M., Leinenbach C. (2013). Stress recovery behaviour of an Fe–Mn–Si–Cr–Ni–VC shape memory alloy used for prestressing. Smart Mater.Struct..

[15] Lee W., Weber B., Leinenbach C. (2015). Recovery stress formation in a restrained Fe–Mn–Si-based shape memory alloy used for prestressing or mechanical joining. Constr. Build. Mater..

[16] Maji A.K., Negret I. (1998). Smart prestressing with shape-memory alloy. J. Eng. Mech..

[17] Dong Z., Klotz U.E., Leinenbach C., Bergamini A., Czaderski C., Motavalli M. (2009). A Novel Fe-Mn-Si Shape Memory Alloy With Improved Shape Recovery Properties by VC Precipitation. Adv. Eng. Mater..

[18] Shahverdi M., Czaderski C., Motavalli M. (2016). Iron-based shape memory alloys for prestressed near-surface mounted strengthening of reinforced concrete beams. Constr. Build. Mater..

[19] Hong K.-N., Yeon Y.-M., Shim W.-B., Kim D.-H. (2020). Recovery Behavior of Fe-Based Shape Memory Alloys Under Different Restraints. Appl. Sci..

[20] Fontana M.G. (2005). Corrosion Engineering.

[21] Jones D.A. (1992). Principles and Prevention of Corrosion.

[22] Uhlig H.H., King C. (1972). Corrosion and corrosion control. J. Electrochem. Soc..

[23] Kim D., Kim Y., Oak J.-J., Lee J., Park C.H., Lee W., Park Y. (2020). Effect of Ni, C and Ti Addition on Shape Recovery Behavior and the Mechanical Properties of Fe-17Mn-5Si-5Cr Shape Memory Alloys. Korean J. Met. Mater..

[24] Amaral S., Müller I. (1999). Passivation of pure iron in alkaline solution containing silicate and sulphate—Galvanostatic and Potentiostatic studies. Corros. Sci..

[25] Bera S., Rangarajan S., Narasimhan S. (2000). Electrochemical passivation of iron alloys and the film characterisation by XPS. Corros. Sci..

[26] Hancock P., Mayne J. (1959). The inhibition of the corrosion of iron in neutral and alkaline solutions. I. J. Appl. Chem..

[27] Haupt S., Strehblow H. (1987). Corrosion, layer formation, and oxide reduction of passive iron in alkaline solution: A combined electrochemical and surface analytical study. Langmuir.

[28] Grosvenor A., Kobe B., Biesinger M., McIntyre N. (2004). Investigation of multiplet splitting of Fe 2p XPS spectra and bonding in iron compounds. Surf. Interface Anal..

[29] Yamashita T., Hayes P. (2008). Analysis of XPS spectra of Fe^2+^ and Fe^3+^ ions in oxide materials. Appl. Surf. Sci..

[30] Biesinger M.C., Payne B.P., Grosvenor A.P., Lau L.W., Gerson A.R., Smart R.S.C. (2011). Resolving surface chemical states in XPS analysis of first row transition metals, oxides and hydroxides: Cr, Mn, Fe, Co and Ni. Appl. Surf. Sci..

[31] Fajardo S., Llorente I., Jiménez J.A., Bastidas J., Bastidas D.M. (2019). Effect of Mn additions on the corrosion behaviour of TWIP Fe-Mn-Al-Si austenitic steel in chloride solution. Corros. Sci..

[32] Kim M.J., Kim J.G. (2015). Effect of manganese on the corrosion behavior of low carbon steel in 10 wt.% sulfuric acid. Int. J. Electrochem. Sci..

[33] Nguyen T.D., Zhang J., Young D.J. (2015). Effects of cerium and manganese on corrosion of Fe–Cr and Fe–Cr–Ni alloys in Ar–20CO_2_ and Ar–20CO_2_–20H_2_O gases at 650 °C. Corros. Sci..

[34] Wilson P., Chen Z. (2007). The effect of manganese and chromium on surface oxidation products formed during batch annealing of low carbon steel strip. Corros. Sci..

